# Stigma and discrimination against transgender men in Bhutan

**DOI:** 10.1371/journal.pone.0287745

**Published:** 2023-07-20

**Authors:** Vinita Saxena, Audrey Xu, Kinley Kinley, Tashi Tsheten, Tenzin Gyeltshen, Tashi Tobgay, Tae Young Zajkowski, Willi McFarland, Lekey Khandu

**Affiliations:** 1 University of California Los Angeles, Los Angeles, CA, United States of America; 2 Stanford University, Stanford, CA, United States of America; 3 Department of Public Health, National HIV, AIDS and STIs Control Program, Ministry of Health, Thimphu, Bhutan; 4 Queer Voices of Bhutan, LGBTIQ Network of Bhutan, Thimphu, Bhutan; 5 Pride Bhutan, LGBTIQ Network of Bhutan, Thimphu Bhutan; 6 Institute of Health Partners, Thimphu, Bhutan; 7 University of California San Diego, La Jolla, CA, United States of America; 8 San Francisco Department of Public Health, San Francisco, CA, United States of America; 9 Department of Public Health, National HIV, AIDS and STIs Control Program, Thimphu, Bhutan; Universidade do Estado do Rio de Janeiro, BRAZIL

## Abstract

**Background:**

While transgender people worldwide face high rates of stigma and discrimination, there are few studies of transgender men (also “trans men”) in Asia. We measured the prevalence of, and factors associated with, stigma and discrimination faced by trans men in Bhutan to bring visibility to their experiences and inform health and social policy changes.

**Methods:**

This cross-sectional survey was conducted in nine regions in Bhutan from November 2019 to January 2020. A total of 124 trans men were recruited using a hybrid venue-based and peer-referral approach. Data were collected using an interviewer-administered questionnaire. Multivariate logistic regression characterized associations with experiencing stigma and discrimination when accessing health services.

**Findings:**

Participants were young (48.0% 18–24 years) and 48.4% had migrated from a rural to an urban area. The majority (95.2%) experienced stigma because people knew or thought they were trans men. Associations with frequent experiences of stigma were living with their partner as a couple (adjusted odds ratio [AOR] 3.07, 95% CI 1.27–7.44) and being unemployed or a student (3.22, 1.44–7.19). Nearly half (47.6%) said they experienced discrimination when accessing health care because people knew or thought they were a trans man; this experience was associated with migration (2.42, 1.08–5.39) and having >15 trans men in their social network (3.73, 1.69–8.26). Most (94.4%) experienced verbal violence, 10.5% experienced physical violence, and 4.8% experienced sexual violence.

**Interpretation:**

Our study found high rates of stigma, discrimination, and interpersonal violence due to being a trans man in Bhutan. Findings highlight the urgent need for strengthening laws and regulations to protect the rights of transgender persons, particularly when accessing health services, recognizing partnerships, and preventing violence in public spaces.

## Introduction

Stigma, discrimination, and violence have been documented among sexual and gender minority people in many parts of the world, ranging from daily micro-aggressions to verbal abuse, physical assault, rape, and death [1]. Discrimination against transgender persons results in denial of academic and other economic opportunities [2]. Transgender men (also “trans men”) are people whose sex is assigned female at birth but self-identify as men. Trans men also encounter abuse and harassment due to laws that aim to regulate public behavior and decency, including combating cross-dressing or imitation of the other sex [3].

Experiences of stigma, discrimination, and violence may prevent trans men’s access to health and social welfare services. Compared to cisgender men, trans men in the United States are less likely to have health insurance and a usual source of health care [4]. Compared to cisgender men, they also experience a higher prevalence of health conditions such as emphysema, liver disease, ulcer, sleep disorders, and poor mental health [5]. The stigma and discrimination against transgender people can lead to hesitation in seeking health and social services, resulting in unmet physical and mental health needs. Unmet mental health needs in turn can lead to high rates of depression and suicide among trans men [6].

According to UNAIDS and the laws in Bhutan, there is no criminalization of transgender people, sex work, or same-sex sexual acts in private [7]. Bhutan’s constitution protects transgender persons for acts such as rape, domestic violence, and sexual harassment in the workplace regardless of gender. However, laws do not explicitly protect LGBTQ+ individuals from discrimination on the basis of sexual orientation, gender identity, or gender expression. In addition, there are no legal explicit legal rights for transgender people to access gender-affirming care, marry, or change government identity documents. Pride Bhutan and Queer Voices of Bhutan, two civil society organizations advocating for LGBTQ+ rights, are not legally registered organizations [8]. It should be recognized that the legal and cultural situation in Bhutan is rapidly changing [9].

Unfortunately, there is very little scientific research published on trans men worldwide, and even less on trans men in Asia [10–13]. Further, to our knowledge, no studies in Asia have measured the prevalence of experiences of stigma, discrimination, and violence experienced by trans men. Therefore, we took the opportunity to ask questions in these domains in the first survey conducted for trans men in Bhutan.

The goals of the current report are to document the presence of stigma and discrimination experienced by trans men in Bhutan and identify factors associated with this stigma and discrimination. We further undertake to present the prevalence of different types of interpersonal violence experienced by trans men, including verbal, physical, and sexual violence. Such data can advocate for policies and practices to support transgender persons in Bhutan and establish a baseline from which to gauge future progress in eliminating stigma, discrimination, and violence toward this vulnerable population.

## Materials and methods

### Overall study design and source population

This study was a cross-sectional survey of trans men conducted in Bhutan from November 2019 to January 2020. Participants were eligible if they were 18 years or older and assigned female sex at birth and self-identified as a gender other than female regardless of hormone use, gender-affirming procedures, sexual orientation, and public gender presentation.

Study locations included nine of Bhutan’s 20 districts (dzongkhag), namely Thimphu, Chhukha (Phuentsholing), Wangdue Phodrang, Sarpang, Paro, Samdrup-Jongkhar, Mongar, Punakha, and Bumthang. The study area covered 64% of the nation’s population and 82% of the urban population. The largest and most urban districts are the capital Thimphu and Chhukha (Phuentsholing), the latter bordering India. These nine districts are located in the three major regions of Bhutan: central, southern border, and the east.

### Survey design and procedures

Details of the sampling and recruitment have been previously described [14] and are summarized here. First, community outreach workers recruited eligible trans men in their social networks to participate and instructed them to refer other eligible trans men from their networks. Second, peer outreach workers also recruited participants from online and physical spaces frequented by LGBTQ+ populations in Bhutan. This resulted in a study sample that was a hybrid of venue, online, and peer-referral recruitment methods. The dates when participants were enrolled are the same as the dates of the survey implementation. Eligible trans men who provided verbal informed consent participated in a structured face-to-face interview. The authors had no access to any information that could identify individuals during or after data collection, and the survey was anonymously conducted. As compensation, participants received cell phone airtime cards of ngultrum 500 (USD $6.79) for survey completion and ngultrum 200 (USD $2.72) for each peer referral.

### Measures

The structured questionnaire was brief and collected demographic information, use of services, and indicator questions on experiences of stigma and discrimination. These indicators are comparable with frameworks and definitions previously used for measuring stigma and discrimination among transgender populations [15]. Translated from the Dzongkha language, the question about stigma was generally asking about experience as a trans man, namely, “Have you experienced stigma because people knew or thought you are a trans man?” The question about discrimination referred to experience with accessing healthcare, namely, “Have you experienced discrimination when accessing health services because people knew or thought you are a trans man?” The answer choices for these two questions ranged from “often,” “sometimes,” and “never.” Participants were also asked if they experienced violence (verbal, physical, sexual) due to being trans men. Occupations labeled as “entertainment” included employment in bars, karaoke clubs, and “drayangs” where dances are performed for customers. These occupations do not explicitly include sex work, although such venues are often locations where sex workers find clients [16]. The questionnaire also included questions on the level of outness about being transgender and the participants’ social network size of other trans men.

### Statistical analysis

Descriptive analysis characterized the demographic profile of the study population and the prevalence of experiences of stigma, discrimination, and violence. We further examined correlates of stigma and discrimination. For correlates of stigma experience, we dichotomized the outcome to be “often” vs. “sometimes/never” due to any experience reported by the vast majority of respondents (i.e., “often” or “sometimes” was reported by 95.2%). For correlates of discrimination when accessing health care, we dichotomized the outcome as “often/sometimes” vs. “never” due to a more balanced distribution of having experienced any such discrimination (i.e., “often” or “sometimes” was reported by 47.6%). We first performed bivariate analysis using the chi-square test to identify potential associations with experiences of stigma and discrimination. Multivariate logistic regression analysis was conducted to characterize independent associations with stigma and discrimination, considering the inclusion of variables associated with these outcomes at p<0.2 in bivariate analysis. Final models retained those variables with p<0.1, while considering only those with p<0.05 as significant. The final models are adjusted for the other variables included. Of note, age, education, and other demographic variables were not associated with stigma or discrimination, nor did they confound any of the associations with the variables included in the final model. Statistical analysis was conducted using StataSE 17.

### Ethics

This study was approved by the Research Ethics Board of Health (REBH) in Bhutan (Ref. No. REBH/Approval/2019/051). Oral consent was obtained from participants.

## Results

Table 1 provides descriptive statistics of this population. A total of 124 trans men were recruited. Participants were young, 48.0% aged 18–24 years, ranging from 18 to 37 years overall. All participants were assigned female sex at birth and currently identified as trans men. Historical terms for transgender persons in Dzongkha, the official language of Bhutan, were generally not used by participants of this study. Most participants (99.2%) were heterosexual, and all participants were born in Bhutan. The majority of participants (98.4%) had an education of middle secondary school and above, and 68.3% had an education level of high school and above. Among participants, 35.5% were living with a partner as if married, and 62.9% were single and never married. One-third (33.9%) held salaried jobs, one-third (33.9%) were students, 28.2% were unemployed, and 4.0% worked in entertainment jobs. Participants’ current residences were 65.3% urban and 34.7% non-urban with nearly half (48.4%) having migrated from non-urban to urban areas.

**Table 1:** Demographic characteristics of trans men in Bhutan, 2020 (N = 124).

Characteristics	N	%
Age group (years)		
18–24	59	48.0
25–37	64	52.0
Sex assigned at birth		
Male	0	0
Female	124	100
Intersex, other, don’t know	0	0
Gender Identity		
Male	0	0
Female	0	0
Trans woman	0	0
Trans man	124	100
Don’t know, other	0	0
Sexual identity		
Straight, heterosexual	123	99.2
Gay	0	0
Bisexual	0	0
Lesbian	0	0
Transmen	1	0.8
Other	0	0
Ethnicity		
Bhutanese	124	100
Non-Bhutanese	0	0
Education		
No education	0	0
Primary	2	1.6
Middle secondary school (grade 7–10)	37	30.1
Higher secondary school (grade 11–12)	79	64.2
University	5	4.1
Marital status		
Living together, not officially married	44	35.5
Single never married	78	62.9
Divorced	2	1.6
Widowed	0	0
Other	0	0
Occupation		
Salaried	42	33.9
Entertainment	5	4.0
Student	42	33.9
Unemployed	35	28.2
Region of birth		
Urban	23	18.6
Non-urban	101	81.5
Current residence		
Urban	81	65.3
Non-urban	43	34.7
Migration		
Non-urban to urban	60	48.4
Urban to non-urban	2	1.6
No migration	62	50.0

*Categories do not always add up to the total due to missing data; percentages shown are among respondents.

Table 2 describes the stigma and discrimination experiences of trans men. The majority (85.5%) stated that many people know they are trans men. Concerning social networks, 51.2% knew more than 15 other trans men. More than half (58.9%) of participants reported often experiencing stigma due to being a trans man, while 3.2% reported never experiencing such stigma. In terms of experiencing discrimination when accessing health services because people thought or knew they were a trans man, 5.7% reported often, 41.9% sometimes, and 45.2% never experienced such discrimination. Most participants (94.4%) experienced verbal violence, 10.5% experienced physical violence, and 4.8% experienced sexual violence due to being a trans man.

**Table 2:** Stigma and discrimination experiences of trans men in Bhutan, 2020 (N = 124).

Question	N*	%
Do people know you are a trans man?		
No one knows this about me	1	0.8
Only a few friends/families know	15	12.1
Many people know	106	85.5
Don’t know	2	1.6
How many other trans men do you know?		
1–15	60	48.8
>15	63	51.2
Have you experienced stigma because people knew or thought you are a trans man?		
Often	73	58.9
Sometimes	45	36.3
Never	4	3.2
Don’t know	2	1.6
Have you experienced discrimination when accessing health services because people knew or thought you are a trans man?		
Often	7	5.7
Sometimes	52	41.9
Never	56	45.2
Don’t know	9	7.3
Have you experienced violence because people knew or thought you are a trans man? (Multiple responses allowed)		
None	7	5.6
Verbal	117	94.4
Physical	13	10.5
Sexual	6	4.8

*Categories do not always add up to the total due to missing data; percentages shown are among respondents.

Table 3 shows the prevalence of stigma and discrimination by different characteristics of trans men. Often experiencing trans-related stigma was significantly higher for individuals who were living with a partner as if married (77.3%, p = 0.002) compared to individuals who are single. By occupation, often experiencing stigma was higher among those who worked in entertainment (80.0%), were students (69.1%), or were unemployed (68.6%, p = 0.009). Often experiencing stigma was more frequent among trans men whose current residence was in an urban area (66.7%, p = 0.015). Experiencing any discrimination was higher among trans men who were living with a partner (65.9%, p = 0.002), with current urban residence (55.6%, p = 0.015), who migrated (61.3%, p = 0.002), and who knew more than 15 trans men (65.1%, p<0.001).

**Table 3:** Associations with stigma and discrimination among trans men in Bhutan, 2020 (N = 124).

	Often experienced stigma, N = 73 (58.9%)		Any discrimination in accessing health services, N = 59 (47.6%)	
Variables		p-value		p-value
Age group (years)		0.353		0.948
18–24	32 (54.2)		28 (47.5)	
25–37	40 (62.5)		30 (46.9)	
Education		0.472		0.354
Middle secondary and below	21 (53.9)		16 (41.0)	
Secondary school and above	51 (60.7)		42 (50.0)	
Marital Status		0.002		0.002
Living together, not officially married	34 (77.3)		29 (65.9)	
Single (includes divorced)	39 (48.8)		30 (37.5)	
Occupation		0.009		0.263
Salaried	16 (38.1)		15 (35.7)	
Entertainment	4 (80.0)		2 (40.0)	
Student	29 (69.1)		23 (54.8)	
Unemployed	24 (68.6)		19 (54.3)	
Region of birth		0.104		0.369
Urban	17 (73.9)		9 (39.1)	
Non-urban	56 (55.5)		50 (49.5)	
Current residence		0.015		0.015
Urban	54 (66.7)		45 (55.6)	
Non-urban	19 (44.2)		14 (32.6)	
Migration		0.362		0.002
Urban/non-urban migration	39 (62.9)		38 (61.3)	
No migration	34 (54.8)		21 (33.9)	
Do people know you are a trans man?		0.191		0.172
No one knows this about me	0 (0.0)		0 (0.0)	
Only a few friends/families know	8 (53.3)		10 (66.7)	
Many people know	65 (61.3)		49 (46.2)	
How many other trans men do you know?		0.338		
1–15	33 (55.0)		18 (30.0)	<0.001
>15	40 (63.5)		41 (65.1)	

*Categories do not always add up to the total due to missing data, percentages are among respondents.

Table 4 shows independent associations for often experiencing stigma and experiencing any discrimination among trans men in Bhutan. Those who were living with a partner as married had 3.07 times the odds of often experiencing stigma as compared to those who did not live with a partner (95% CI 1.27–7.44). Those who were students or unemployed had 3.22 times the odds of often experiencing stigma as compared to those with paying jobs (95% CI 1.44–7.19). Trans men who had migrated experienced 2.42 times the odds of experiencing any discrimination in accessing health care compared to those who had not migrated (95% CI 1.08–5.39). Those who knew more than 15 trans men had 3.73 times the odds of experiencing any discrimination when accessing health services compared to those who knew fewer trans men (95% CI 1.69–8.26).

**Table 4:** Multivariate analysis: Independent associations with stigma and discrimination experiences of trans men in Bhutan, 2020 (N = 124).

Outcome	Predictors	Adjusted* odds ratio (95% confidence interval)	p-value
**Often experienced stigma**	Living with partner Student/unemployed Current urban residence	3.07 (1.27–7.44) 3.22 (1.44–7.19) 2.19 (0.96–4.99)	0.013 0.004 0.064
**Experienced any discrimination in accessing healthcare**	Living with partner Any urban/non-urban migration Trans men’s social network >15	2.11 (0.91–4.89) 2.42 (1.08–5.39) 3.73 (1.69–8.26)	0.081 0.031 0.001

*Adjusted for the other variables listed in each model.

## Discussion

Our study measured very high levels of stigma, discrimination, and violence due to being trans male in Bhutan. Our data corroborate high levels of stigma and discrimination suggested by the few available studies of trans men worldwide [10–13,17]. Many of these studies were qualitative and therefore direct comparison to the prevalence found in our data is not possible. Nonetheless, a consistent pattern of high prevalence of stigma and discrimination is evident. For example, experiences of stigma were common in a study of trans men in Puerto Rico, with components described as structural, interpersonal, and individual [10]. Similarly, in Nigeria, trans men expressed frequent stigma when accessing HIV services for being transgender [11]. In this Nigerian trans male population, respondents specifically mentioned struggling with gender identity disclosure, facing reduced sensitivity when interacting with healthcare providers, and not receiving gender-inclusive HIV services [11]. Participants in a study with trans men in Uganda reported stigma related to disclosing their gender identity and HIV status, including at healthcare facilities, leading to decreased access to services [12]. A study in the United States found that trans men were less likely to seek medical care due to discrimination [13]. At least one quantitative study, from Peru, reported higher levels of discrimination experienced by trans men from healthcare providers (69.0%) than we found in Bhutan (47.6%) [17]. To our knowledge, our study is the first to measure stigma and discrimination among trans men in Asia. Also, to our knowledge, it is the largest study of trans men in Asia, with 124 participants.

Although the sample size was small in absolute terms, we were able to identify significant correlates of stigma and discrimination. For example, living with a partner was associated with higher odds of experiencing stigma and discrimination. As same-sex marriage is not yet officially recognized in Bhutan, marriage for transgender persons remains unclear. We therefore take the response of “living together” when asked about their marital status as a proxy for being married. We hypothesize that the association with stigma and discrimination may be due to the increased visibility a co-habiting couple may face in public. Current urban residence was also associated with experiencing higher levels of stigma and migrating to an urban area was associated with increased discrimination. We hypothesize a similar reason may explain these findings in that trans men in an urban area may be living more openly as transgender and therefore more easily targeted by stigma and discrimination. The finding that having more trans men in their social network being associated with increased discrimination may also support this increased visibility hypothesis; that is, being part of the community may entail higher visibility as transgender. Moreover, the questions themselves were phrased with the implication that the experienced occurred because people knew or thought they were trans men. However, because this study is cross-sectional, there may be a reversal of this cause and effect. That is, trans men experiencing stigma and discrimination may move to urban areas for greater anonymity, and they may live with their partner and seek out other trans men for increased safety or support after having faced stigma and discrimination. Our finding that trans men who were students or unemployed did not report higher levels of discrimination was unexpected because discrimination from academic and employment opportunities have been noted in previous studies of transgender persons [18,19].

Experiencing stigma and discrimination due to being trans men can also cause multiple adverse mental health effects. One study found that interpersonal stigma, which includes interpersonal violence and everyday stigma, was associated with poor mental health [20]. These researchers also found that experiencing everyday discrimination was associated with depression, anxiety, post-traumatic stress disorder, and self-injury in trans men. A Canadian study that investigated the mental health of transgender youth, including transmasculine youth, posited that high rates of mental health problems might be attributed to stigma and related social and familial struggles [21]. Another study in New Zealand found trans men had higher prevalence of anxiety and depression diagnoses than trans women [22]. The researchers attributed the findings to trans men experiencing additional stressors, including higher reported rates of sexual abuse, domestic violence, and discrimination when accessing healthcare and employment services. Trans men might also use substances as a way of coping with the stigma and discrimination they experience. One study found that 27.6% of trans men reported substance use as a coping technique for experiencing stigma [23]. Experiences of stigma and discrimination by transgender persons could also lead to eating disorders. A study of sexual and gender minority populations found transgender and gender non-conforming adults more likely to report current or past eating disorders compared to gay men [24]. Of note, other evidence finds trans men with protective factors to potentially mitigate mental health disorders. Such protective factors for trans men are being in a committed relationship, older age, increased resilience, and family connectedness [20–21]. Fostering these protective factors could provide increased support and benefit to trans men facing multiple levels of stigma and discrimination.

This study has limitations that must be noted. First, as mentioned above, the cross-sectional design does not allow for determining the temporal cause-and-effect relationships between correlations. Second, because the survey was brief, it included only a few questions on experiences of stigma and discrimination providing limited context and detail. We acknowledge the need for primary theoretical research on the context and measurement of stigma and discrimination for sexual and gender minority populations in Bhutan. Third, the representativeness of our sample is not known. We note that the young age of the sample is consistent with Bhutan’s age structure [25]. However, the proportions in our sample that are urban and with secondary education appear higher than among the general population. Whether this is true of all trans men or only for those whom we could reach and who were willing to participate in our study is not known. We highlight factors of our study that may strengthen inference. Recruitment was not conducted through clinics or services, as is often the case for studies of trans men [22,26,27]. Moreover, recruitment included hybrid methods that helped further diversify the sample by reaching trans men who are less visible (i.e., through peer referral) as well as those who may be less connected to peers (i.e., through online and physical venues). Although the sample size is small, 124 participants, it should be noted that this is estimated to constitute a large proportion of the reachable transgender population estimated at 0.06% of adult men in Bhutan [14]. Finally, we also note that our survey is one of the largest of trans men to date. We know of only one online-recruited survey of greater sample size [28] and one other peer-referral plus venue-based recruited survey of equal size [29], both predominantly of trans men living in the United States.

In summary, our data point to very high rates of stigma and discrimination experienced by trans men in Bhutan, in line with many other parts of the world [10–13]. These experiences appear associated with factors that increase their visibility in public where they may face multiple contexts for stigma and discrimination, including when seeking health services. Our data speak to the need to strengthen anti-discrimination legislation to include gender identity and sexual orientation and recognize committed partnerships. While dismaying to see such high levels of physical and emotional violence directed at this community, we believe documentation of their high prevalence can help advocate for these policies and laws to protect Bhutanese trans men to make their lives healthier and happier.
